# Sustainable Dental and Periodontal Practice: A Narrative Review on the 4R-Framework—Reduce, Reuse, Rethink, Recycle—And Waste Management Rationalization

**DOI:** 10.3390/dj13090392

**Published:** 2025-08-28

**Authors:** Federica Di Spirito, Francesco Giordano, Maria Pia Di Palo, Giuseppina De Benedetto, Leonardo Aulisio, Giovanni Boccia

**Affiliations:** Department of Medicine, Surgery and Dentistry, University of Salerno, 84081 Baronissi, Italy; frgiordano@unisa.it (F.G.); giusydb15@gmail.com (G.D.B.); laulisio@unisa.it (L.A.); gboccia@unisa.it (G.B.)

**Keywords:** green dentistry, sustainable dentistry, periodontology, environmental impact, waste management, air pollution, water conservation, life cycle assessment, digital dentistry, teledentistry

## Abstract

**Background/Objectives**: While dentistry plays a critical role in promoting oral health, it also contributes significantly to environmental degradation through high energy consumption, water usage, and reliance on disposable, non-recyclable materials. Periodontology, in particular, involves resource-intensive procedures such as full-mouth disinfection, frequent surgical interventions, and aerosol-generating instrumentation. The aim of the present narrative review is to synthesize current knowledge and delineate feasible, evidence-informed strategies to operationalize sustainability across the full spectrum of periodontal treatment settings. **Methods**: The electronic search of the present narrative review was performed across PubMed/MEDLINE, Web of Science, BioMed Central, Scopus, CINAHL, and Cochrane Library databases. **Results**: The review identified actionable sustainability strategies across pre-workplace (e.g., eco-conscious procurement and transport reduction), workplace (e.g., energy- and water-saving technologies, digital workflows, and pollution control), and waste management (e.g., reuse protocols, recycling, and sustainable material selection). Particular emphasis was placed on the role of dental education, life cycle assessments, and digital innovations. **Conclusions**: The transition toward sustainable periodontology requires the adoption of evidence-based practices and leveraging digital innovation to reduce the environmental impact while maintaining high standards of care.

## 1. Introduction

Despite its fundamental mission to promote individual and public health, the healthcare sector paradoxically contributes substantially to environmental degradation. Globally, it is responsible for approximately 4.4% of total greenhouse gas (GHG) emissions [1].

**Figure 1:**
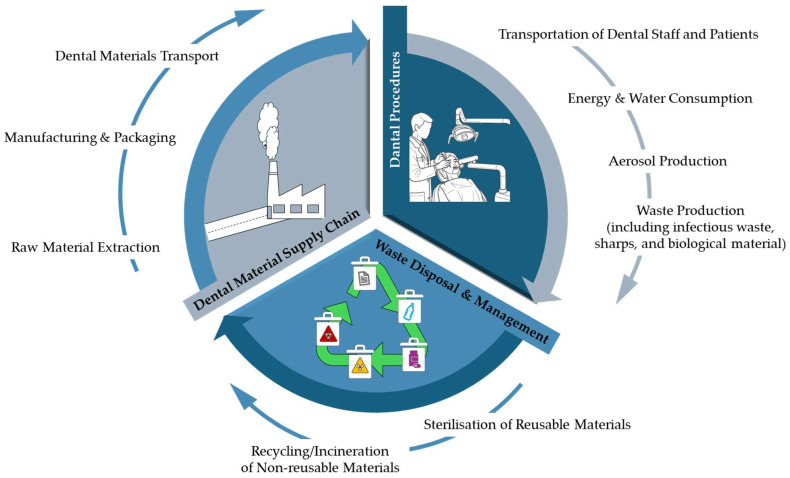
Dentistry environmental impact and overall ecological footprint: from dental materials supply chain to dental procedures and waste disposal and management.

Dentistry, though a relatively small sector, significantly impacts the environment through its reliance on the dental material supply chain, energy-intensive technologies, excessive water consumption, patient and staff travel, and the widespread use of single-use and non-recyclable materials [2], as shown in Figure 1.

In response to these challenges, the concept of green dentistry—also referred to as sustainable or eco-friendly dentistry—has emerged. It represents a paradigm that integrates environmental stewardship with clinical safety and effectiveness. Its overarching goals are to reduce the ecological footprint of oral healthcare by optimizing material and energy use, minimizing waste and pollution, and aligning clinical operations with global planetary health agendas, including the United Nations Sustainable Development Goals and national climate action plans [2,3].

In detail, from nonsurgical periodontal therapy to regenerative surgeries, periodontal care contributes to the environmental burden through the use of ultrasonic scalers, surgical drapes, disposable instruments, and irrigation systems [4,5]. Yet, it also holds opportunities to implement greener alternatives, such as reusable metal instruments, water-efficient ultrasonic devices, aerosol-reducing hand techniques, and digital periodontal charting [3,4,5].

In addition, as a prevention-oriented specialty, periodontology can also promote long-term oral and systemic health, indirectly reducing the need for high-carbon interventions [6,7,8].

However, the implementation of sustainable strategies in periodontology remains limited. Contributing factors include structural, financial, and educational barriers. Many dental professionals lack formal training in environmental sustainability, and the financial burden of adopting eco-efficient technologies—such as high-efficiency heating ventilation and air conditioning (HVAC) systems, dry vacuum suction, or solar infrastructure—remains a limiting factor [2]. The situation is further exacerbated by the absence of standardized metrics for evaluating environmental performance in periodontal care, such as carbon footprints of common procedures or data on surgical consumables’ Life Cycle Assessment (LCA). LCA is a methodological framework used to assess the environmental impacts associated with all stages of a product’s life, from material extraction to disposal [2,3,9].

Compounding these challenges are legacy infection prevention and control (IPAC) protocols that, while critical for safety, often rely on environmentally costly practices. These include extensive use of sterilization cycles, chemical disinfectants, and disposable personal protective equipment (PPE), many of which are not biodegradable [2,10]. The COVID−19 pandemic further entrenched reliance on single-use items, but now presents an opportunity to reassess protocols through a dual lens of infection control and environmental responsibility [11,12].

Ultimately, sustainable transformation in periodontal care—and dentistry at large—will require an integrated, multistakeholder effort. Regulatory bodies must incentivize green procurement, product transparency, and emissions reporting [2]. Academic institutions should embed sustainability in dental education, emphasizing its relevance not only in general practice but also in specialist training programs like periodontology [13,14]. At the same time, robust research is needed to map environmental hotspots in periodontal workflows, from patient triage to post-operative care, and to develop evidence-based recommendations for greener clinical pathways [3].

Despite its conceptual promise, green dentistry adoption remains fragmented and under-resourced. To bridge this gap, clinicians, policymakers, manufacturers, and educators must collaborate to translate sustainability principles into actionable strategies within periodontal care [3,15]. Against this backdrop, the aim of the present narrative review is to synthesize current knowledge and delineate feasible, evidence-informed strategies to operationalize sustainability across the full spectrum of periodontal treatment settings.

## 2. Materials and Methods

On 10 July 2025, an electronic search was started across PubMed/MEDLINE, Web of Science, BioMed Central, Scopus, CINAHL, and Cochrane Library databases to retrieve pertinent English research about sustainability in oral healthcare, such as original human studies and systematic reviews without restriction on year of publication. A manual search in the reference lists of relevant records retrieved through the electronic search was performed. Studies on oral disease prevention and health promotion, minimization of environmental harm, systems-based sustainability, equity and justice, as well as evidence-based innovation in oral healthcare were included. Exclusion criteria were: non-English records, narrative reviews, short communications, and commentaries.

In the searched databases, the filter “English language” was applied, and the keywords used for the search were:

- (“green dentistry” OR “sustainable dentistry” OR “eco-friendly dentistry” OR “environmentally sustainable oral healthcare” OR “sustainable oral healthcare” OR “sustainable dental practice”) AND
- (“environmental impact” OR “carbon footprint” OR “greenhouse gas emissions” OR “environmental sustainability” OR “waste reduction” OR “pollution reduction”) AND
- (“disposable materials” OR “reusable products” OR “recycling” OR “water conservation” OR “energy conservation” OR “renewable energy” OR “life cycle assessment” OR “LCA”) AND
- (“dentistry” OR “oral healthcare” OR “dental practice” OR “dental clinics” OR “periodontal management” OR “periodontal therapy” OR “periodontology”)

The relevant records were collected by three independent reviewers (F.D.S., M.P.D.P., and G.D.B.) and managed using Mendeley Reference Manager software (version 2.80.1).

Data from the selected studies were extracted and recorded using a specialized extraction form created in Microsoft Excel 2019 (Microsoft Corporation, Redmond, WA, USA).

The data collected from the relevant studies were qualitatively synthesized in the present narrative review.

The evidence-based strategies reported in the present review are drawn from a broad spectrum of evidence sources, including international and national policy regulations, clinical guidelines, expert consensus documents, systematic reviews, and observational and cross-sectional studies. Where available, priority was given to sources with higher levels of evidence and rigorous methodologies (e.g., systematic reviews, consensus guidelines), while innovative or emerging practices were included when supported by early clinical reports.

## 3. Results

### 3.1. Sustainability in Health Care and Environmental Impact of Oral Healthcare

Healthcare organizations (HCOs) have a central role in global public health, but contribute significantly to global pollution, notably through greenhouse gas (GHG) emissions and medical waste. The U.S. health sector alone accounts for approximately 6–8% of national GHG emissions and 25% of global health sector emissions, primarily from Scope 3 supply chain activities [2,3,4,5]. Despite the sector’s environmental burden, sustainability practices remain fragmented and largely unregulated. Misconceptions around sustainability’s strategic importance and a lack of organizational expertise among health system leadership have slowed progress. Nonetheless, momentum is building through regulatory and investor demands for transparency and action on environmental, social, and governance (ESG) criteria [10,11,12,13,14]. The environmental footprint of oral healthcare is particularly multifactorial, spanning energy use, patient and professional travel, single-use materials, and supply chain emissions [16]. Dental procedures—ranging from routine check-ups to complex restorations—generate significant GHGs, largely due to operational demands and material intensity. A standard dental examination is estimated to produce 5.5 kg carbon dioxide equivalents (kgCO_2_e), with restorative treatments reaching up to 15 kgCO_2_e, depending on clinical complexity [17].

Infection control protocols drive reliance on disposables, many of which are non-recyclable due to contamination. Consequently, clinical waste generated by dentistry contributes to landfill and incineration burdens. Furthermore, the production and distribution of dental materials (e.g., composites, resins, alloys) entail environmental costs, including fossil fuel use, water consumption, and emission of hazardous compounds. These indirect impacts are especially pronounced in disjointed or poorly regulated supply chains. LCA has increasingly emphasized the need for more sustainable workflows and material choices in oral healthcare [17].

### 3.2. Sustainability as a Core Business Strategy in Health and Oral Care and Principles of Sustainable Oral Healthcare

Integrating sustainability into the business strategy of HCOs is crucial for advancing environmental justice and health equity. Vulnerable communities—disproportionately affected by climate change—often lack the infrastructure to adapt or respond, despite contributing minimally to global emissions [16,17]. Health systems must take a proactive role in mitigating environmental harm, not only as a public health obligation but also as a matter of strategic risk management and social accountability [18,19,20].

Sustainability should be embedded within dentistry as an additional principle of value-based care. Reducing over-treatment, optimizing material use, and embedding sustainability in reimbursement and quality metrics are avenues for aligning ecological and clinical goals [16,17]. Five foundational principles guide the transition to sustainable dentistry [17]:

1. Disease prevention and health promotion: the most sustainable care is care that is not needed. By preventing caries and periodontal disease, reliance on resource-intensive interventions can be reduced.
2. Minimization of environmental harm: dentistry must reduce emissions, eliminate avoidable waste, and shift to low-impact materials. Innovations such as teledentistry, energy-efficient equipment, and green procurement can reduce the carbon burden.
3. Systems-based sustainability: sustainability must be integrated across institutional domains, including education, policy, procurement, infrastructure, and clinical operations.
4. Equity and justice: sustainability efforts must not compromise access to care or exacerbate disparities. Policies should support equitable distribution of sustainable technologies and services.
5. Evidence-based innovation: interventions should be guided by robust environmental data and clinical outcomes, avoiding superficial “greenwashing” and emphasizing continuous quality improvement [17].

### 3.3. Sustainable Dental and Periodontal Practice: 4R Framework—Reduce, Reuse, Rethink, and Recycle

In the pursuit of environmentally sustainable dentistry, the 4R framework—Reuse, Recycle, Rethink, and Reduce—provides an essential foundation for minimizing the ecological footprint of oral healthcare systems.

Reduce underscores the need to minimize resource consumption in dental practice. This principle supports the rational use of electricity, water, and clinical supplies through interventions such as low-energy lighting systems, dry vacuum pumps, and precise inventory management to prevent overordering. At a systemic level, reduction also involves emphasizing disease prevention and health promotion to decrease the need for invasive, material-intensive treatments in the first place [17]. Together, the 4Rs encapsulate a holistic and actionable framework for aligning dental care with the goals of planetary health and climate resilience.

Reuse involves the systematic replacement of single-use items with sterilizable alternatives such as stainless-steel trays, reusable suction tips, cloth bibs, and autoclavable impression trays. This shift not only curbs the generation of plastic waste but also yields long-term economic benefits, provided that strict sterilization protocols are maintained to uphold infection control standards [1,17,21]. 

Rethink challenges traditional models of care and procurement by urging dental professionals and organizations to reconsider how materials are sourced, used, and disposed of. This involves adopting life-cycle thinking, prioritizing sustainable procurement, and integrating environmental criteria into clinical decision-making. For instance, when several clinically equivalent options are available, practitioners are encouraged to choose products with minimal packaging, verified life-cycle assessments, or those manufactured under environmentally responsible conditions [17]. Rethinking also extends to care delivery, where digital technologies such as teledentistry and virtual consultations reduce emissions from travel and facilitate more resource-efficient workflows [22].

Recycle, as the second pillar, emphasizes the importance of effective waste segregation within clinical environments. Dental practices generate considerable volumes of non-hazardous materials—such as paper, cardboard, plastics, and aluminum—that can be diverted from landfills through well-implemented recycling programs and staff training. Nonetheless, recycling in dentistry faces constraints due to the presence of biohazardous contamination, which limits the recyclability of many clinical items [17].

### 3.4. Sustainable Dental and Periodontal Practice: Strategies

**Figure 2:**
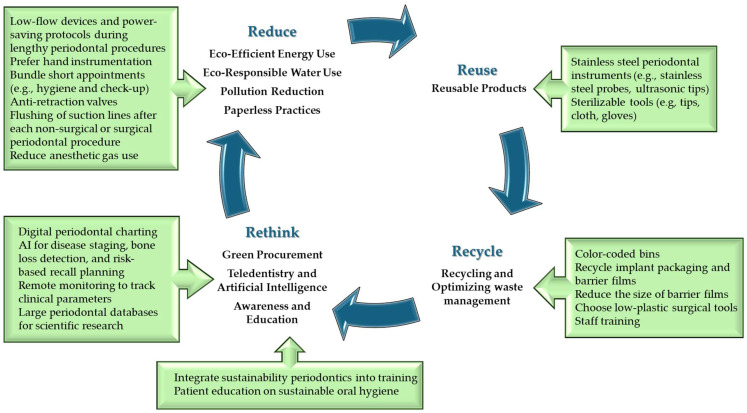
4R framework in dental and periodontal practice and related strategies.

Specific strategies aligning with the 4R framework—Reuse, Recycle, Rethink, and Reduce—have been detailed below (Figure 2).

#### 3.4.1. Eco-Efficient Energy Use and Eco-Responsible Water Use

Energy consumption in dentistry arises from the use of lighting systems, air conditioning, sterilization equipment, and digital technologies. Substituting traditional lighting with light-emitting diode (LED) and installing motion-based occupancy sensors helps lower electricity use. Optimization of HVAC systems, either through regular maintenance or replacement with high-efficiency units, is also encouraged [17,18]. Dental practices adopting renewable energy sources—such as photovoltaic panels—demonstrate reduced greenhouse gas emissions. Incorporating sustainability guidelines like those outlined in the “Green Impact Dentistry” toolkit further promotes energy-conscious decision-making [18].

In periodontics, the cumulative energy consumption extends from the initial diagnostic to the treatment and maintenance phases. For example, diagnostic radiographic imaging acquisition (e.g., periapical, panoramic radiographs), which is essential for periodontal bone loss assessment, requires electricity from image acquisition to digital processing and storage. While diagnostic imaging itself cannot forgo electricity, its carbon footprint can be mitigated by transitioning to renewable electricity, which could substantially decrease the indirect radiation footprint [19].

Additionally, digitalization of periodontal charting minimizes energy use related to printing periodontal charts. A fully digital workflow, including electronic patient medical histories and clinical documentation, not only supports the elimination of routine printing activities, and directly contributes to lower electricity consumption, reducing the use of energy-intensive devices as printers, but also limits deforestation and reduces the volume of waste generated in dental settings [3].

Periodontal therapy procedures such as full-mouth disinfection (FMD) and supportive periodontal therapy (SPT) typically require prolonged chair time and the extensive use of ultrasonic scalers (0.79 kWh per day estimated, dental suction systems (0.29 kWh per day), compressor (2.04 kWh per day), operatory lighting (1.20 kWh per day), and autoclave systems (9.68 kWh per day). The use of ultrasonic devices with energy-saving modes, intelligent operatory scheduling, and automated device shut-off systems can help reduce cumulative electricity consumption during high-frequency procedures [20].

Despite the availability of sustainable energy, its adoption in the dental setting remains limited. A questionnaire-based study of Veress et al. (2023) [23] showed that only 1.02% of 98 dentists operated a dental office with its own energy production. From an eco-efficient energy use perspective, transitioning to renewable energy sources or generating your own energy is beneficial [23]. 

Dental settings are high water consumers due to procedures that require irrigation, suction, sterilization, and sanitation. A central strategy for conservation is the replacement of wet-ring vacuum systems with dry vacuum alternatives, which can reduce water consumption by up to 85% [24,25]. Additional measures include the use of autoclaves with water recirculation systems, routine leak detection, and the installation of faucet aerators or flow restrictors. These technologies not only conserve water but also reduce operational costs and ecological impact. Recent discussions also emphasize the need to standardize water-efficient devices across all dental settings, including periodontics, and to include water stewardship in sustainability education initiatives [24,25].

Despite many dental professionals expressing interest in learning more about water quality, studies indicate a persistent gap between environmental awareness and actual water conservation practices [26,27]. For instance, even with over 78% of clinicians showing interest in water quality, routine waterline maintenance protocols or consistent flushing habits are often lacking, particularly among dentists without support staff [27]. Interestingly, while dentists with more experience may perform less frequent cleaning of dental units (e.g., weekly versus daily cleaning by less experienced dentists), those with international training or higher education levels tend to exhibit greater awareness of water quality standards and perform more frequent maintenance [27]. Conversely, younger professionals with fewer years of experience often demonstrate a higher level of knowledge regarding water hygiene regulations [26].

In periodontology, where frequent irrigation, ultrasonic scaling, and surgical procedures are common, specific actions can significantly improve water use efficiency. The rigorous and uniform flushing of suction lines and waterlines after both non-surgical and surgical periodontal therapy for each patient is essential. While optimal flushing durations vary by system and the length of the dental unit’s water network, recommendations suggest flushing for 1–5 min to reduce microbial contamination [27]. It is important to consider that both units connected to public water supplies and units relying on reservoir bottles should incorporate anti-retraction valves and flush after each treatment to minimize the risk of contamination [26]. Instead, the flushing of handpieces should ideally be fitted with anti-reflux valves or flushing mechanisms and always flushed for 20–30 s after each patient is treated [27]. This seemingly small action, when consistently applied, contributes significantly to overall water conservation.

Sustainable water use in periodontology should also consider indirect sources of water consumption, such as the production of PPE, including dental clothing. Traditional cotton-based clothing has a high environmental footprint due to its intensive water demands during cultivation and processing. In fact, the production of cotton contributes significantly to water scarcity and ozone depletion [19]. 

#### 3.4.2. Air Pollution Reduction

Dental practices contribute to environmental air pollution through mercury from dental amalgam, volatile chemical disinfectants, aerosol generation, and anesthetic gas emissions. Amalgam separators—now widely mandated—are essential in preventing mercury contamination of wastewater. Additionally, the replacement of high-volatile organic compounds (VOC) disinfectants with environmentally safer alternatives and the implementation of filtration and suction systems reduce air and surface pollution [12,28,29].

While indoor air pollution resulting from dental procedures, especially those involving high-speed instruments such as ultrasonic scalers used in non-surgical periodontal therapy, has been thoroughly investigated to control cross-infection [12,29,30,31]. However, a substantial lack of data regarding the contribution of dental practices to outdoor air pollution limits the current knowledge on the impact of periodontics on outdoor air pollution. Duane et al. (2017) [9] assessed the carbon footprint of various dental procedures in England, showing that examinations (5.50 kg carbon dioxide equivalents (kgCO_2_e) per individual procedure), radiographs (5.50 kgCO_2_e), and non-surgical periodontal treatments (6.53 kgCO_2_e) individually have a relatively low carbon footprint [9]. However, due to their high frequency across the dental healthcare system and also due to the longer duration of the periodontal treatment and related maintenance, these low-emission procedures cumulatively contribute significantly to the overall environmental burden. In fact, examinations that have the lowest carbon footprint if assessed individually, have the highest rate of the overall carbon footprint of dental procedures in England, accounting for 27.08% (181.433 tonnes of CO_2_e (TCO_2_e) per year) of the total footprint of dental services [9]. Non-surgical periodontal treatments account for 13.5% of total emissions (90.087 TCO_2_e) and radiographs for 6.41% (42.930 TCO_2_e) [9]. In contrast, the extractions for the periodontally hopeless teeth registered the lowest proportion on the overall carbon footprint of dental procedures (3.54%, 8.58 kgCO_2_e per individual procedure, and 23.744 TCO_2_e per year) [9].

Furthermore, the addition of nitrous oxide to dental procedures can generate up to 119 kgCO_2_e per item (5.829 TCO_2_e per year) [9]. The anesthetic gases’ high-impact emissions could be mitigated using industrial scrubbing technologies, which are already common in hospital settings to extract or neutralize gases before they are released into the atmosphere [9].

Beyond emissions related to periodontal procedures, dental travel emerges as the major source of pollution linked to dental care, contributing an estimated 64.5% of dentistry’s total carbon footprint in England, releasing more than 443 tonnes of nitrogen oxides and 22 tonnes of fine particulate matter annually [20,32]. Patient travel accounts for 31.1%, staff commuting 30.3%, and work-related staff travel 3.1%, which significantly impacts outdoor air pollution [32]. The short duration of many periodontal appointments, such as dental hygiene or check-up visits, creates a paradox: while the procedures themselves are low-impact, the carbon emissions associated with the patient’s travel are high. 

To address this, periodontitis and dental hygienists should bundle appointments (e.g., combining family visits or scheduling non-surgical periodontal treatments together with a check-up), and where feasible, employ single-visit digital workflows (e.g., intraoral scanning for prosthetics) to reduce repeated patient travel [32]. Another key strategy is the integration of telemedicine, which has already shown success in the periodontal management of pediatric subjects in rural healthcare contexts through remote consultations facilitated by hygienists and intraoral cameras [32]. Such models have demonstrated reduced travel needs, improved access, and lower overall emissions [32]. From a supply chain perspective, partnering with local dental laboratories or coordinating delivery with staff commutes can also cut down travel-related emissions [32]. 

#### 3.4.3. Reusable Products

The replacement of single-use disposables with durable, sterilizable alternatives is a key focus in sustainable dental operations. Reusable options such as stainless steel suction tips, glass dappen dishes, and cloth gowns have demonstrated lower environmental and financial costs over time, despite the initial investments in sterilization infrastructure [33,34]. Studies comparing lifecycle impacts have confirmed that reusables, when maintained correctly, outperform disposables in terms of waste generation, carbon emissions, and cost-effectiveness [33,34]. 

In periodontics, metal periodontal probes, reusable ultrasonic tips, and surgical instruments significantly cut down the single-use plastic burden. However, the plastic and nitrile waste in the dental setting represents about 34% and 15% of the overall solid dental waste, respectively. Furthermore, gloves are the second most frequently disposed of type of waste in a dental setting (26%) [35]. Thus, beyond the issue of disposable instruments, the high volume of plastic and nitrile waste from single-use protective equipment, especially gloves, represents a major obstacle to sustainable periodontics.

During the public health crises of the Ebola outbreak and the SARS-CoV−2 pandemic, new decontamination strategies for the high risk of infection and reuse of gloves due to logistical challenges in supply chains, because of high demand, were investigated [36,37]. Research conducted under these crisis conditions, while primarily focused on maintaining barrier integrity and pathogen inactivation, provides valuable insights that could be useful to future sustainable dentistry. Studies have investigated the compatibility of different glove materials (e.g., latex, nitrile, vinyl) with various decontamination methods [36,37]. Findings suggest that latex gloves, particularly surgical grades due to their thickness, often exhibit superior resistance to barrier compromise compared to vinyl gloves, which tend to fail across multiple decontamination methods [37]. While some methods, like quats, have been shown to degrade mechanical and thermal properties of certain glove types (e.g., nitrile and vinyl), others, such as ultraviolet, ethanol, heat, and steam, demonstrated greater material compatibility over multiple disinfection cycles for up to 10–20 uses [36].

Specifically for the dental setting, the Danish ReGlove project has piloted a circular system for reusable nitrile gloves in hospitals and dental clinics, including dedicated collection, washing, and redistribution infrastructure. Supported by the Danish Environmental Protection Agency, the initiative demonstrated that gloves can be safely reused up to 15 times, with validated LCA showing lower environmental impact after just two uses. This model offers a compelling pathway to reduce glove-related waste and carbon emissions in clinical dentistry (https://www.dti.dk/services/reglove-reusable-nitrile-gloves-for-healthcare/44636, accessed on 12 July 2025). 

#### 3.4.4. Paperless Practices

Digitization in dentistry has emerged as a strategic tool for sustainability. The transition to electronic health records (EHR), cloud-based radiographic storage, digital intraoral scanning, and paperless patient consent systems significantly decreases paper use. These technologies improve clinical efficiency while also reducing the consumption of ink, toner, and physical storage space. Dental schools are increasingly integrating digital workflow competencies into their curricula, framing paperless practices as both environmentally and professionally advantageous [3].

In periodontics, digital periodontal charting and virtual documentation of treatment plans streamline communication and reduce printouts. The adoption of digitization in periodontics should be useful to reduce paper waste in dental settings, which represents about 12% of the overall solid dental waste, as reported in the case study of Richardson et al. (2016) [35].

Beyond the immediate waste reductions, paperless practices in periodontics support one of the five foundational principles that guide the transition to sustainable dentistry: evidence-based innovation, which should be guided by robust environmental data and clinical outcomes [17]. Clinical periodontal research has been confined to academic facilities, treating less than 1% of the population in the U.S., the remaining 99% occurs in private dental practices [38]. To bridge the research-practice gap and offer more relevant and responsive evidence and innovations, the Practice-Based Research Network (PBRN) was introduced [38]. The paperless practices in periodontics should allow the extensive collection of periodontal patient data digitally, representing a potential new large database for scientific research [38]. This allows for the collection and analysis of real-world patient periodontal data from several private dental practices [38]. 

#### 3.4.5. Recycling and Optimizing Waste Management

Effective recycling protocols within dental practices are hindered by the co-existence of infectious and non-infectious waste. Interventions such as color-coded and clearly labeled bins, supported by staff training and compliance audits, have improved waste diversion rates [39,40]. Barriers remain due to the treatment requirements of clinical waste, which often prevent recycling. Nonetheless, practices that recycle outer packaging, cardboard, sterilization wraps, and non-contaminated plastics have shown reductions in incineration volume. Advocacy efforts like the Green Impact awards recognize progress in dental waste segregation and recycling [39,40,41]. 

Although international guidelines on medical waste share core principles, such as the strict segregation and mandatory treatment of infectious materials, differences exist in how these rules are defined, enforced, and operationalized across regions (Table 1). These differences can affect the feasibility of implementing certain sustainability practices.

Despite these variations, the overarching principle remains: sustainable waste management in periodontology must operate within the boundaries of legal and infection control frameworks, targeting non-contaminated waste streams as the primary area of intervention.

In periodontal care, surgical kit packaging, implant component wrappings, and barrier films are the main sources of waste that could be improved by both reducing their size and improving their recycling workflows. A survey of dental practices found that while standard office paper is widely recycled, the paper and plastic components of autoclave bags are often overlooked, despite being suitable for common recycling programs [41]. This suggests a clear area for immediate improvement. 

#### 3.4.6. Green Procurement

Environmentally conscious procurement focuses on reducing upstream environmental impacts by selecting sustainable products. Prioritized items include biodegradable and autoclavable tools (e.g., stainless-steel impression trays), reusable cloth drapes, and packaging-free supplies when multiple clinically equivalent options are available [43,44]. LCAs are increasingly employed to inform purchasing choices. Market pressure and accreditation systems are encouraging suppliers to disclose environmental impact data and participate in circular economy programs. Efforts such as bulk purchasing and the preference for local suppliers further reduce transportation-related emissions [32].

Periodontal departments can prioritize suppliers offering sustainable surgical drapes and regenerative biomaterials with minimal plastic content. 

Particular attention should also be given to the environmental footprint of daily oral hygiene aids, such as interdental brushes and floss, which are essential for preventing periodontal disease [6]. A recent LCA analysis reveals considerable variation in environmental impacts among different inter-dental cleaning aids. Bamboo-handled inter-dental brushes and replaceable head inter-dental brushes showed lower footprints, while plastic floss picks were associated with a higher environmental footprint [45]. Although clinical effectiveness remains paramount, these findings support integrating sustainability criteria into product selection for periodontal maintenance [45]. Crucially, as these devices are not only integral to professional therapy but also to home-based periodontal maintenance, it becomes fundamental for dentists to educate their patients on these sustainable choices.

#### 3.4.7. Teledentistry and Artificial Intelligence

Teledentistry and Artificial Intelligence (AI)-based digital technologies are pivotal in reducing the environmental footprint of oral healthcare by streamlining service delivery, limiting unnecessary travel, and optimizing clinical workflows. By allowing for virtual triage, remote consultations, digital health promotion, and postoperative follow-up, teledentistry reduces carbon emissions associated with physical appointments while improving care accessibility [46,47]. This transition is particularly important given that transportation-related emissions are among the most significant contributors to healthcare’s overall carbon burden [48].

Telehealth’s environmental advantages have been demonstrated across multiple health systems. In Spain, digital consultations saved over 6700 tons of CO_2_ in just one year, and in Sweden, telerehabilitation prevented over 82,000 km of travel, translating into a substantial carbon offset [49,50]. These findings are echoed in dental settings, where teledentistry applications, such as remote monitoring, asynchronous diagnostics, and cloud-based treatment planning, significantly reduce the need for resource-intensive in-person visits. Despite these advantages, the adoption of these technologies is hampered by limited awareness, digital infrastructure, and integration into dental education [46,51].

In periodontics, AI has revolutionized periodontal therapy, now assisting with radiographic interpretation, allowing periodontal bone loss detection, disease staging, outcome prediction, and the development of individualized treatment plans, enabling risk-based recall scheduling, and surgical planning [52,53].

Remote monitoring platforms can support ongoing maintenance therapy, allowing clinicians to track clinical parameters, plaque control, and healing progression without requiring in-person visits. This is particularly impactful given that supportive periodontal therapy often involves frequent recall appointments. The use of intraoral cameras, mobile apps, and patient-uploaded images, analyzed through AI algorithms, enables the early detection of inflammation, plaque accumulation, and potential recurrence of disease [54,55].

AI systems further enhance the sustainability of periodontal practice by increasing diagnostic efficiency and reducing unnecessary interventions. AI algorithms have demonstrated high accuracy in detecting radiographic bone loss, staging periodontitis, and predicting treatment outcomes. Convolutional Neural Networks and models like Faster R-CNN, U-Net, and VGG-16 can segment alveolar bone structures, identify periodontal defects, and classify disease severity with precision [56,57,58]. These tools reduce variability in diagnosis and facilitate evidence-based, individualized treatment planning. Moreover, AI-based risk stratification enables tailored recall intervals, optimizing chair time and resource use while reducing patient visits [59,60].

From an operational perspective, AI can support sustainability through workflow automation, improving appointment scheduling, inventory control, and patient communication. Predictive analytics can inform procurement needs based on usage trends, helping minimize material waste. Digital periodontal charting and cloud-based patient records also reduce paper consumption and facilitate interprofessional collaboration [61].

In conclusion, AI, particularly through machine learning and deep learning neural networks, offers a complementary and potentially transformative avenue for sustainability in dentistry [53]. Despite these advantages, it is crucial to acknowledge the environmental cost of AI itself. The environmental cost of training large AI models is substantial, with the carbon emissions from training some deep learning models surpassing the lifetime emissions of a car [53]. Sustainable AI development must therefore include the use of smaller, hybrid models, energy-efficient hardware, and federated learning strategies—where training occurs locally and only updated parameters are shared—to protect data privacy and minimize computational emissions. Transparency and trustworthiness are also essential. The rise of “explainable AI” seeks to demystify how AI systems reach clinical decisions by visualizing the most influential variables in model predictions. These tools are essential for integrating AI into evidence-based dentistry and ensuring that clinical decisions remain accountable, equitable, and ethically sound [53]. To mitigate these issues, dental institutions should prioritize energy-efficient AI models, federated learning systems, and hardware optimization to align with sustainability goals [53].

Table 2 summarizes the available data on the estimated contribution of periodontal intervention to the total environmental footprint of dentistry, as well as the impact of dentistry-related activities and resource use. The estimated impact of sustainable alternatives in dentistry was also highlighted.

#### 3.4.8. Awareness and Education

Sustainable transformation within dentistry is predicated on a foundational realignment of professional ethics, values, and behaviors toward planetary health. One of the most impactful strategies to catalyze this transformation is the integration of environmental sustainability into dental education at all levels. Embedding sustainability into undergraduate and postgraduate curricula, onboarding protocols, and continuing professional development (CPD) could integrate ecological responsibility within daily clinical practice [13,63].

The AMEE Consensus Statement underscores the imperative of cultivating a sustainability mindset within the health professions, advocating for a pedagogical model that includes transformative learning, systems thinking, and experiential engagement with real-world ecological dilemmas. It calls for the alignment of educational goals with the Sustainable Development Goals (SDGs), particularly those related to good health and well-being (SDG 3) and climate action (SDG 13), encouraging health professionals to act as change agents across the spectrum of healthcare delivery [13].

Parallelly, the scoping review by Martin et al. [14] reveals substantial gaps in both professional and public awareness of sustainability in dentistry, identifying education as a critical leverage point. The review emphasizes that current dental training often lacks structured content on environmental stewardship, leading to fragmented understanding and inconsistent implementation of sustainable practices in clinical settings. Barriers include a paucity of sustainability-related competencies in accreditation standards, limited access to pedagogical resources, and inadequate faculty development in this domain [14].

Evidence from pilot programs and curriculum reviews suggests that effective sustainability education in dentistry should include practical training modules (e.g., sustainable procurement, waste segregation), visual prompts (e.g., infographics in clinical zones), and simulation-based learning. Furthermore, green accreditation frameworks for clinics and departments serve to reinforce theoretical knowledge with institutional behavior, embedding sustainability into the cultural fabric of dental education and practice [15].

Beyond professional education, community outreach and patient education materials are effective in disseminating sustainability principles. By engaging the broader public in discussions around environmental responsibility—such as the environmental costs of certain materials, the value of prevention, and sustainable oral hygiene products—dental professionals can contribute to a wider cultural shift toward ecological consciousness [14].

Ultimately, a paradigm shift in dental education requires systemic support, including curricular reform, interprofessional collaboration, institutional leadership, and research investment. Such an integrated approach will not only address the current awareness gaps but also foster a generation of practitioners equipped to lead sustainability transitions in oral healthcare [13,14,63].

In periodontics, education should emphasize eco-conscious instrumentation selection, aerosol-minimizing techniques, and sustainable biomaterial usage, as well as the integration of AI-driven diagnostic tools for early and precise detection, digital treatment planning to reduce solid waste, and the application of teledentistry for remote monitoring and travel reduction. Pilot programs should integrate visual prompts and simulation-based modules to reinforce theoretical learning with behavioral change and technological adoption.

Community outreach and patient education campaigns on sustainable oral hygiene (e.g., avoiding microplastic-releasing mouthwashes or floss) further expand ecological awareness. In this context, dental educators—periodontists, hygienists, and general dentists—play a crucial role in extending sustainability education directly to patients during periodontal maintenance and oral hygiene instruction. International campaigns such as “Save 90 a day!” by the Eco-Dentistry Association^®^ (https://ecodentistry.org/green-dental-patients/save-90-day-patients/, accessed online on 13 July 2025) illustrate a practical and impactful approach to make patients aware of environmental conservation, pointing out that the average person wastes about 90 glasses of water a day by leaving the tap running while brushing their teeth, which, when compared to the population, translates into billions of glasses of clean water wasted every day (https://ecodentistry.org/green-dental-patients/save-90-day-patients/, accessed online on 13 July 2025). 

Below is Table 3, summarizing the main strategies for sustainable dentistry.

## 4. Discussion

Recent contributions to the literature have explored the foundational elements of sustainability within dental practices, emphasizing the interplay between environmental health and dental healthcare delivery. International health authorities have underscored the urgent need to address the health impacts of climate change, particularly given the paradox that healthcare systems themselves are significant contributors to global greenhouse gas emissions (https://www.who.int/news/item/27-11-2023-global-health-community-calls-for-urgent-action-on-climate-and-health-at-cop28, accessed on 15 July 2025) [64]. The estimated total greenhouse gas emission of the dental services within the National Healthcare System (NHS) in England (2013–2014) amounted to 675 kilotons of carbon dioxide equivalent, representing up to 3% of the England NHS’s overall carbon footprint [9]. Although dentistry accounts for a relatively modest fraction of this sector, it presents both responsibility and opportunity for environmental impact. In fact, this proportion (about 3%) is comparable to other outpatient medical specialties but higher than certain preventive services on a per-procedure basis due to high patient travel emissions (about 64% of dental footprint) and reliance on single-use plastics [65]. In contrast, acute hospital care generates most of its emissions from energy use, pharmaceuticals, and inpatient service, while travel (patient, staff, and visitors) represents only about 10% of the NHS’s total carbon emissions [65]. Thus, while dentistry’s impact is smaller than high-resource hospital departments (e.g., surgery, intensive care), it is disproportionately influenced by travel (similarly to mental health services) and consumable waste [9,65], offering distinct opportunities for targeted emission reduction.

A tripartite model has been proposed to structure sustainability within dental practice, addressing the pre-workplace domain (including procurement and commuting), the workplace itself (with focus on clinical activities and energy use), and waste management (covering disposal and post-treatment processes). Periodontics, with its procedural intensity and reliance on multiple operatories, surgical instruments, and disposables, is particularly well-suited for sustainability innovations within each domain as described below.

### 4.1. Sustainability in Periodontal Practice: Pre-Workplace Domain

Efforts to reduce emissions from transportation include scheduling family appointments jointly, encouraging active and public transport among patients and staff, and modifying infrastructure to support bicycle commuting and low-emission travel modes [32]. For instance, transitioning from multiple short appointments to comprehensive full-mouth treatments—when clinically appropriate—can minimize transport-related emissions and resource redundancy [32].

Environmentally responsible procurement further enhances sustainability by prioritizing biodegradable materials, reusable goods, and suppliers with transparent ecological policies. The growing use of LCA, essential in evaluating the comparative sustainability of reusable versus single-use items and clinical procedures, in purchasing decisions, alongside institutional pressures for circular economy participation, is transforming procurement norms [33,34]. In periodontology, material selection must follow clinical guidelines and evidence-based criteria. However, when clinically equivalent products are available, LCAs could inform the selection of regenerative biomaterials, guided tissue regeneration membranes, and implant packaging systems, prioritizing those with lower embedded energy, recyclable, and biodegradable configurations.

Attention has also been directed toward procurement strategies, with recommendations for local sourcing, reduced packaging, and bulk purchasing. 

### 4.2. Sustainability in Periodontal Practice: Workplace Domain

Subsequent research has shifted attention to the internal workings of dental settings, also implying periodontal practice, particularly focusing on water and energy use, and organizational culture. Those utilizing renewable energy sources—such as photovoltaic panels—have demonstrated notable reductions in emissions. Moreover, sustainability frameworks like the “Green Impact Dentistry” toolkit offer structured approaches to environmentally conscious decision-making [3].

Water consumption in dental practices remains high due to irrigation, suction, and sterilization procedures. Notably, dry vacuum systems have shown up to 85% reductions in water use compared to traditional wet-ring alternatives. Complementary technologies such as water-recirculating autoclaves, leak detection systems, and flow restrictors enhance conservation efforts and yield financial savings [24,25]. Sustainable water management in periodontology requires addressing also indirect water consumption from PPE production, preferring alternatives like bamboo or recycled ocean plastic fabrics for reduced environmental impact [19].

The energy demands of lighting, air conditioning, sterilization equipment, and digital technologies are well-documented. Mitigation strategies have included replacing conventional lighting with LEDs, installing motion sensors, and optimizing HVAC systems through maintenance and high-efficiency upgrades [17,18]. Periodontal operating rooms, often requiring prolonged surgical times, should integrate motion-sensor lighting, automated HVAC zoning, and energy-efficient sterilizers.

The spatial organization of dental facilities also plays a role in sustainability. Efficient use of space, including the consolidation of treatment areas and the reduction of underutilized zones, leads to lower energy use. Strategies such as smart thermostats and improved insulation have further enhanced energy efficiency [18].

Pollution control has emerged as a growing priority, driven by awareness of the ecological toxicity associated with mercury from amalgam, high-VOC disinfectants, and aerosols. The mandatory use of amalgam separators, safer chemical alternatives, and high-performance filtration and suction systems has contributed to measurable improvements in air and water quality [12,28,29]. Periodontal procedures often generate high aerosol loads; thus, the use of high-volume evacuation, high-efficiency particulate air filtration units, and reduced-aerosol instruments should be prioritized [12].

Instead, to minimize travel-related outdoor air pollution, periodontists and dental hygienists should bundle appointments (e.g., combining family visits or scheduling non-surgical periodontal treatments with check-ups) and if feasible, implementing single-visit digital workflows. Furthermore, telemedicine proves highly effective, especially for patients in rural areas, cut down travel and improve access. Optimizing the supply chain by collaborating with local dental laboratories or coordinating deliveries with staff commutes also helps reduce transport-related emissions [32].

Meanwhile, internal interventions—such as improving building insulation, integrating solar power, enhancing digital record-keeping, and adopting telehealth systems—offer practices opportunities to reduce their environmental footprint directly.

Refs. [12,46,51,53,66] Digital technologies, including teledentistry and AI, offer significant sustainability benefits in periodontal care by reducing travel-related emissions, optimizing workflows, and supporting remote maintenance therapy [45,50,52]. However, barriers such as limited infrastructure, digital literacy, and concerns over data security continue to hinder widespread adoption [45,50]. Additionally, the environmental cost of training large AI models must be addressed through strategies like federated learning and energy-efficient algorithms [52]. These tools, when responsibly implemented, can help align periodontal practice with broader sustainability goals.

### 4.3. Sustainability in Periodontal Practice: Waste Management

Waste reduction through minimalist packaging, reusable containers, and instrument sterilization is widely promoted. The environmental drawbacks of incinerating medical waste have led to calls for improved segregation protocols and expanded recycling programs [39,40,41]. In periodontics, durable alternatives to disposables—such as stainless steel suction tips—and custom trays and surgical packs could be redesigned for modular reuse and to minimize non-contaminated waste. LCAs continue to support the superiority of reusables under appropriate sterilization protocols [41]. The selection of sterilizable, reusable materials over disposable counterparts, when feasible and safe, represents a cornerstone of sustainable procurement practice applicable to periodontics.

As part of ongoing organizational reform, dental practices are also revisiting equipment longevity, upcycling options, and the use of low-toxicity alternatives to standard disinfectants. These shifts are supported by early studies into the health and environmental impacts of various dental chemicals [67,68].

Furthermore, by highlighting eco-friendly alternatives for oral hygiene aids and explaining their benefits, periodontists and dental hygienists can empower patients to adopt more environmentally responsible daily oral hygiene routines, extending the practice’s commitment to sustainability far beyond the dental setting [41,45].

Recycling implementation in dental and periodontal settings may be favored by practical interventions [39,40]. The paper and plastic components of autoclave bags were identified as the prime target for increased recycling efforts or even replacement with more sustainable alternatives. Based on broader controls, even small adjustments, such as using cloth sterilization wrappings instead of disposable ones or recycling paper from sterilization pouches, can produce substantial cumulative benefits in busy clinical environments [41].

### 4.4. Policy and Recommendations for Sustainable Dental and Periodontal Practice

Oral health should be embedded in initiatives such as the World Health Organization’s Health in Climate Change program, the United Nations Climate and Health Declaration, and national climate action plans (https://www.who.int/initiatives/who-wmo-joint-climate-and-health-programme, accessed on 15 July 2025) [64].

National health strategies should include sustainability objectives that align with planetary health commitments, and regulatory frameworks should incentivize the procurement of low-carbon dental materials and mandate environmental disclosures. However, it should also be taken into account that while the principles of sustainable dentistry are universal, their practical adoption is influenced by regional factors [14,69]. Regulatory frameworks governing infection control, waste segregation, and the use of hazardous materials differ in scope and enforcement between countries, reflecting variations in legal systems, public health priorities, and available infrastructure [14], as shown in Table 1. In high-income regions, strict legislation and robust waste management systems can facilitate compliance but may also introduce cost and administrative burdens that discourage innovation toward greener practices [69]. Conversely, in low- and middle-income settings, limited infrastructure or economic resources may hinder the implementation of advanced sustainability measures, even where awareness exists [69].

These disparities highlight the importance of tailoring sustainability strategies to local regulatory and socioeconomic contexts. Mechanisms such as green public procurement and environmental performance benchmarking are essential to normalize sustainable practice. Accountability can be enforced through public reporting of greenhouse gas emissions, life cycle assessment data, and environmental, social, and governance progress [3,11,15,18,35].

Dental professionals must embrace preventive strategies and low-impact treatment options, actively participate in energy and waste reduction, and educate patients on sustainable practices. In periodontics, this includes prioritizing non-surgical interventions when clinically appropriate [17], clustering treatment sessions to reduce operatory time [12] and associated emissions, using hand instruments and closed irrigation systems to reduce aerosol generation, and selecting reusable probes, curettes, and ultrasonic tips in both maintenance and surgical care [33,34]. 

Instead, to minimize water consumption, acquiring knowledge of own dental unit’s specific characteristics is essential to understand the flushing time required and significantly reduce water waste after every patient. The same level of awareness should be obtained regarding optimal flushing times that apply to the use of handpieces and turbines after periodontal treatments [27]. 

To mitigate also the indirect hidden water cost of dental clothing, periodontists should explore alternatives such as bamboo fiber fabrics or clothing made from recycled ocean plastic, which offer a lower environmental impact in terms of both water usage and greenhouse gas emissions [19].

Manufacturers and suppliers should prioritize eco-friendly materials, transparent life-cycle disclosures, and recyclable packaging. In the context of periodontics, this includes advancing the development of low-plastic regenerative membranes, biodegradable sutures, minimally packaged instruments, and reusable surgical textiles, supported by standardized environmental performance indicators [19].

Although the exploration of PPE reuse and decontamination during public health crises caused by Ebola and SARS-CoV-2 has opened up new frontiers in waste management [37,38], it is essential to consider the preliminary nature of these findings. Infection control remains the top priority in dental and periodontal settings. The use of clinically validated single-use products is the current standard of care, offering a proven and reliable method of preventing cross-contamination. Recently studied innovations aimed at greater sustainability must first demonstrate their ability to meet or exceed these rigorous standards of safety and sterility before being adopted in medical routine practice.

Even if further comprehensive studies are needed, the preliminary research on reusable gloves opens to a more eco-friendly dental future, suggesting a potential shift towards reusable or multi-use glove strategies and a substantial reduction of disposable gloves in routine dental care, aligning with the broader goals of sustainable periodontics.

Incentives for green procurement, waste segregation, and climate resilience [16], and, specifically, for periodontal practices adopting energy-optimized ultrasonic devices and paperless digital charting systems should also be considered along with selective PPE protocols, waste stream segregation, and lean inventory models [2,10], especially considering that waste audits in dental education settings have revealed that periodontal simulations produce high volumes of packaging and non-infectious disposables.

Academic institutions are called to integrate sustainability into dental curricula and continuing education, fostering a new generation of environmentally conscious professionals. A core component of sustainability lies in reshaping dental education. Incorporating environmental content into undergraduate and postgraduate programs fosters a culture of ecological responsibility. Consensus statements advocate for sustainability to be central in healthcare curricula, aligned with the Sustainable Development Goals. However, implementation gaps persist due to inadequate accreditation standards, limited pedagogical resources, and insufficient faculty training. Effective educational interventions include hands-on sustainability modules, visual prompts in clinical settings, and simulation-based learning. Institutional commitment, public engagement, and green certifications further reinforce sustainability as a shared value [15]. In periodontal training, specific emphasis should be placed on minimizing plastic waste during surgeries, critical evaluation of material packaging, and sustainable patient communication strategies [13,14,63].

Furthermore, the upload of periodontal patients’ data on digital shared databases should represent a more sustainable pathway for advancing evidence-based care in an eco-friendly periodontics [38]. By leveraging existing clinical infrastructure and routinely collected periodontal digital information, it minimizes the need for resource-intensive research setups and optimizes the use of already-generated data, fostering a continuous cycle of quality improvement with a significantly reduced ecological footprint [38]. 

Parallel responsibilities exist in broader healthcare organizations, where operational sustainability must be supported through leadership endorsement, procurement reforms, and cross-departmental coordination [16]. 

In this context, periodontists, dental hygienists, and national or international societies engaged in reinforcing patient motivation and education for periodontal maintenance can play a pivotal role in advancing environmental sustainability by informing and educating patients about emerging ecological knowledge and ongoing environmental campaigns at local, national, and global levels.

### 4.5. Barriers to Change Towards Sustainable Dentistry

The integration of sustainable practices into dental care is hindered by a complex interaction of economic, regulatory, technical, and behavioral factors, with particular difficulties in resource-constrained settings, where financial barriers and lack of knowledge are most evident [14].

The increasing protocols on infection prevention and control of infectious diseases have led to a higher use of single-use plastics, which account for the bulk of dental solid waste [14,70,71]. Regulatory infection control guidelines are sometimes perceived as conflicting with sustainability goals, creating uncertainty for healthcare providers in the transition to green dentistry [14,72]. Furthermore, sustainable alternatives remain limited, and the high costs of other recycled products, such as paper, create additional barriers [11,73]. Psychological resistance to the reuse of medical and dental devices, like the new frontier of multi-use gloves for medical purposes, also limits potential reductions in waste production [11,37].

Waste recycling in dental practices is hindered by multiple barriers, including the need for additional storage, transportation costs, staff training, perceived safety risks, limited access to recycling facilities, and insufficient public and cultural awareness [14,74,75]. The heterogeneity of medical wastes, particularly in dentistry, where different materials are used, adds complexity to segregation and disposal [14,71].

In many regions, especially in developing economies, medical waste management suffers from inadequate knowledge, negative attitudes, insufficient training, and the absence of regulatory frameworks that distinguish medical waste from household waste [14,75,76,77,78]. A favorable combination of education, infrastructure, and legislation is essential but often absent due to a lack of funding and political priority [14,76,77,78].

Infrastructural deficiencies also play an important role in the lack of easily accessible alternatives to private car travel for dental staff and patients, which can preclude reductions in carbon emissions from dentistry-related transport [32].

In contrast, while innovations such as teledentistry and AI hold promise for reducing the environmental footprint of dental care by decreasing patient travel, optimizing resource use, and improving diagnostic efficiency, their widespread adoption is still limited by several challenges. In many regions, limited internet connectivity, inadequate digital infrastructure, the high initial cost of equipment, as well as concerns regarding data privacy, cybersecurity, and compliance with health information regulations, have limited the integration [46,79]. There is also a lack of training and digital literacy, especially among older healthcare providers, leading to resistance or underutilization of these tools [46,79]. Additionally, the absence of clear guidelines on how to measure and verify the environmental benefits of digital technologies in dentistry represents another barrier [46,79].

Addressing these barriers requires a multifaceted and tailored approach. Overcoming these barriers will require targeted investments in digital infrastructure, standardized protocols for data protection, and dedicated training programs that build both technical competence and confidence in the environmental value of these innovations.

Economic incentives, such as subsidies for sustainable materials or tax relief for waste management improvements, could offset cost barriers, particularly in low- and middle-income countries. In fact, the cost-effectiveness of these eco-friendly interventions depends on the context, with shorter payback periods in high-volume practices or regions with high waste management costs [14]. High-income private clinics may face fewer capital constraints, but barriers can still arise from perceived inconvenience, lack of regulatory alignment, or insufficient evidence on return on investment. In contrast, low-income private practices and underfunded public clinics often operate with limited budgets, ageing infrastructure, and minimal access to specialized waste management or digital tools, making initial adoption of sustainable practices more challenging. In these contexts, prioritizing low-cost, high-impact interventions, such as optimized waste segregation, pooled procurement, shared sterilization facilities, and low-bandwidth teledentistry, can enable incremental progress [14,80,81]. Public clinics may benefit from leveraging governmental procurement channels and infrastructure, whereas private low-income settings may rely more on manufacturer take-back schemes or micro-financing to cover upfront costs. Policy reforms should align infection control regulations with sustainability objectives, encouraging innovation in reusable and safe materials. Investment in infrastructure, including recycling facilities and public transport options, can reduce logistical constraints, while targeted procurement policies [14]. Finally, expanding research into LCA for all dental materials and cost-effectiveness research on teledentistry options will provide the evidence base needed to guide environmentally responsible clinical practice [14,82].

### 4.6. Future Prospects

Given the emerging nature of sustainable dentistry, future research should focus on key areas to accelerate the transition toward greener clinical practices. The path toward a more sustainable dental sector will be shaped by scientific innovation, systems thinking, and interdisciplinary action. In particular, critical topics for future research include: Environmental Toxicology and Periodontal Health.

Future research should prioritize low-carbon alternatives, sustainable clinical protocols, and interventions that promote circularity [2]. In addition, future directions should investigate microplastic exposure through periodontal irrigants, polishing agents, and oral hygiene products commonly used in periodontal maintenance. The presence of micro- and nanoplastics in soft tissues, including the gingiva, could represent a novel avenue of research at the intersection of environmental toxicology and periodontal pathology [83].

- Development of Standardized LCA protocols tailored for the dental setting

Life Cycle Assessment (LCA) remains the most robust tool, yet harmonization of protocols and expansion to include planetary boundaries and ecosystem impacts are needed [2].

In periodontology, future LCAs should specifically address the footprint of regenerative biomaterials, barrier membranes, bone substitutes, and disposable surgical kits used in flap procedures. These interventions, while clinically effective, involve energy-intensive production and packaging processes. Evaluating their environmental impact could foster a shift toward greener alternatives without compromising clinical efficacy.

- New Technologies and Sustainable Innovation

AI and digital health tools are expected to accelerate sustainable change. Predictive models and algorithm-driven treatment planning can reduce over-treatment and streamline care delivery [53]. In periodontics, AI integration into risk-based recall scheduling, bone loss monitoring, and virtual surgical simulation could optimize appointments and reduce unnecessary resource use. However, the energy cost of training AI systems must be considered, and greener algorithm designs (e.g., federated learning) should be prioritized [2].

- Educational Frameworks and Behavioral Change

The transition to net-zero dentistry will depend not only on new technology but also on behavioral and structural changes across the system. The role of dental education remains foundational. Consensus reports recommend embedding environmental sustainability at all levels of health professional training [13]. Periodontology curricula should incorporate modules on green surgery, sustainable instrumentation, and minimally invasive approaches with low material intensity. Clinical decision-making frameworks could include carbon footprint data as part of evidence-based care models Targeted investments, policy alignment, procurement standards, and accreditation metrics must work in synergy. As climate-related health threats escalate, the periodontology community has a responsibility to lead by example, embedding planetary health into daily care and professional ethics.

## 5. Conclusions

The integration of sustainability principles into periodontal practice represents both a professional responsibility and a strategic opportunity to align oral healthcare with global sustainability goals. From procurement to clinical workflows, waste management, and digital innovation, a comprehensive and interdisciplinary approach is essential to reduce the environmental footprint of periodontal care without compromising clinical efficacy. Key strategies include adopting energy- and water-efficient technologies, shifting toward reusable and low-impact materials, and leveraging digital tools such as teledentistry and artificial intelligence to streamline care and reduce resource use. The future of sustainable periodontology will rely on robust life cycle assessments, interdisciplinary collaboration, and system-wide commitment to behavioral, technological, and structural changes.

Education plays a foundational role: institutional support, green procurement incentives, and environmental performance benchmarks are crucial to institutionalizing sustainable practices in dental settings.

As oral health professionals stand at the intersection of patient care and planetary stewardship, periodontists should also promote environmental responsibility within clinical practice and empower patients to adopt sustainable oral health behaviors.

## Figures and Tables

**Table 1:** Summary of regional legal policy governing clinical waste management (competent authority, essential requirement, traceability, transport regulations) and practical concerns.

Region	Sub- Region	Competent Authority	Essential Requirements	Traceability	Transport Regulations	Key Notes	Practical Management and Concern	References
Europe	EU (Member States)	European Commission; national environment/health ministries	Source separation; waste management hierarchy	Mandatory records (varies by country)	ADR; Waste Shipments Regulation	Implementation varies among Member States	Widespread use of two-/three-bin systems (infectious, sharps, general waste); steam sterilization (autoclave) and high-temperature incineration for infectious waste and expired pharmaceuticals	(https://eur-lex.europa.eu/eli/dir/2008/98/oj/eng, accessed on 9 August 2025)
	Non-EU European countries (e.g., UK, Switzerland, Norway)	National/federal environment ministries	Mandatory separation and treatment	National manifest systems	ADR (for international transport)	Similar to EU practices but adapted to the local subregion	Similar to EU practices; higher prevalence of contracts with certified private operators; frequent use of microwave treatment in the UK	(https://www.fedlex.admin.ch/eli/cc/2015/891/en?print=true&version=20250422; https://www.legislation.gov.uk/uksi/2024/1281/made; https://www.legislation.gov.uk/uksi/2012/811/pdfs/uksics_20120811_en.pdf, accessed on 9 August 2025)
Americas	North America (USA, Canada)	EPA; state/provincial agencies	Autoclaving, licensed incineration	Manifest in many states/provinces	DOT/PHMSA (USA); provinci al rules (Canada)	No single strong federal framework	USA: extensive use of centralized facilities for autoclaving/shredding; Canada: often hospital-based treatment followed by disposal in controlled landfills or incinerators	(https://www.cdc.gov/infection-control/hcp/environmental-control/regulated-medical-waste.html; https://www.epa.gov/rcra/model-guidelines-state-medical-waste-management; https://biosecuritycentral.org/resource/requirements-and-protocols/canadian-biosafety-standard/, accessed on 9 August 2025)
	Latin America (e.g., Brazil, Mexico, Argentina)	National health/environment ministries	Separation, sterilization, or incineration	Manifest required in many countries	National rules + ADR if exported	Enforcement varies; infrastructure challenges	Brazil and Mexico: frequent use of dedicated incineration facilities; rural areas still see burial or burning in rudimentary furnaces	(https://ampeid.org/documents/brazil/resolution-conama-no-358-ruling-on-final-disposal-of-waste-originated-from-sanitary-services/; https://conama.mma.gov.br/images/conteudo/CONAMA-ingles.pdf, accessed on 9 August 2025)
Asia	East Asia (Japan, Republic of Korea, China)	National environment; health ministries	Separation, licensed facilities, autoclave/incineration	Manifest mandatory	National transport rules	Generally robust enforcement	Japan/Korea: sealed rigid containers; mandatory autoclaving for infectious waste; incineration for pharmaceuticals and anatomical waste; China: increasing number of provincial sterilization facilities	(https://www.env.go.jp/en/laws/recycle/01.pdf; https://elaw.klri.re.kr/eng_service/lawView.do?hseq=48601&lang=ENG, accessed on 9 August 2025)
	South Asia (India, Bangladesh, Pakistan)	National health/environment ministries	Separation; treatment within 48 h	Manifest mandatory in India	National transport rules	Gap between regulations and actual practice	India: color-coded segregation (yellow, red, blue, black) followed by autoclaving/incineration; in smaller centers, open burning still common	(https://dhr.gov.in/sites/default/files/Bio-medical_Waste_Management_Rules_2016.pdf; https://cpcb.nic.in/uploads/projects/bio-medical-waste/guidelines_healthcare_june_2018.pdf, accessed on 9 August 2025)
	Southeast Asia (e.g., Thailand, Malaysia, Vietnam)	National health/environment ministries	Separation; incineration/autoclave	Manifest not always mandatory	National transport rules	Technical capacity varies	Thailand: in-house hospital incinerators; Malaysia/Vietnam: mix of autoclaving and controlled landfilling; rural areas face major gaps	(https://www.ratchakitcha.soc.go.th/DATA/PDF/2564/A/014/T_0015.PDF; accessed on 9 August 2025) [42]
Africa	North Africa (e.g., Egypt, Morocco, Tunisia)	National health/environment ministries	Separation; management in licensed facilities	Manifest requirements vary	National transport rules	Implementation hindered by limited resources	Egypt: mostly hospital-based incinerators; Morocco: regional plans for mobile autoclaves and centralized collection	(https://faolex.fao.org/docs/pdf/egy4986E.pdf; https://adsero.me/waste-management-executive-regulations-amendments-prime-ministers-decrees-no-1113-and-no-1114-of-2024/?utm_source=chatgpt.com, accessed on 9 August 2025)
	Sub-Saharan Africa (e.g., South Africa, Kenya)	National health/environment ministries	Separation; management in licensed facilities	Manifest requirements vary	National transport rules	Infrastructure gaps; WHO often provides support	South Africa: certified private operators with autoclaving/shredding; Kenya: low-tech incinerators and landfill disposal	(https://faolex.fao.org/docs/pdf/saf138344.pdf; https://www.informea.org/en/legislation/regulations-relating-health-care-waste-management-health-establishments-2014-gn-r-no-375?utm_source=chatgpt.com, accessed on 9 August 2025)

Abbreviations: European Union, “EU”; United Kingdom, “UK”; Agreement for transport of Dangerous goods by Road, “ADR”; United States of America, “USA”; Department of Transportation, “DOT”; Environmental Protection Agency, “EPA”; Pipeline and Hazardous Materials Safety Administration, “PHMSA”; World Health Organization, “WHO”.

**Table 2:** Estimated contribution of periodontal interventions, resource use, and operational activities to the total environmental footprint of dentistry, and potential impact of sustainable alternatives.

Dental Procedure and Activities/Source	Estimated Environmental Impact	Estimated Impact of Sustainable Alternatives
Dental procedure and activities		
Clinical examinations [9]	27.08% of the total carbon footprint of the dental practice 5.50 kgCO_2_e/procedure 181,433 TCO_2_e/year	
Radiographs [9]	6.41% of the total carbon footprint of the dental practice 5.50 kgCO_2_e/procedure 42,930 TCO_2_e/year	
Non-surgical periodontal therapy [9]	13.5% of the total carbon footprint of the dental practice 6.53 kgCO_2_e/procedure 90,087 TCO_2_e/year	
Surgical periodontal therapy	No available data	
Extractions (hopeless teeth) [9]	3.54% of the total carbon footprint of the dental practice 8.58 kgCO_2_e/procedure 23,744 TCO_2_e/year	
Nitrous oxide use [9,62]	0.9% of the total annual carbon footprint emission 119.00 kgCO_2_e/procedure 5829 TCO_2_e/year	Nitrous oxide scavenging system: −71–91% of the waste of indoor nitrous oxide compared to no scavenging system
Travel [9,20,32] (https://gitnux.org/sustainability-in-the-dental-industry-statistics/, accessed on 13 August 2025)	64.5% of the total annual carbon footprint emission: Patient travel 31.1%, Dental staff travel 30.3% Work-related dental staff travel 3.1%	Teledentistry for follow-ups/consultations: −60% travel-related emissions Digital workflows: −15% patient travel-related emissions
Procurement (materials and services) [9]	19.0% of the total annual carbon footprint emission	
Source		
Energy and Gas [9] (https://gitnux.org/sustainability-in-the-dental-industry-statistics/, accessed on 13 August 2025)	15.3% of the total annual carbon footprint emission Autoclave 9.68 kWh/day Compressor: 2.04 kWh/day Operatory lighting: 1.20 kWh/day Ultrasonic scaler: 0.79 kWh/day Dental suction system: 0.29 kWh/day	LED lighting: −75% electricity use compared to halogen Eco-friendly sterilization chemicals: −85% of high-volatile organic compounds emissions
Water [9,24,25] (https://gitnux.org/sustainability-in-the-dental-industry-statistics/, accessed on 13 August 2025)	0.1% of the total annual carbon footprint emission	Water-saving dental units: −20–30% water use Dry vacuum: −85% water use compared to wet-ring vacuum Digital impressions: −50% of waste compared to traditional impressions
Waste [9,35] (https://gitnux.org/sustainability-in-the-dental-industry-statistics/, accessed on 13 August 2025)	0.2% of the total annual carbon footprint emission 2–5 tons annually Plastic waste: about 34% Nitrile waste: about 15% Paper waste: about 12% of the total solid dental waste	Paperless digital records: –10,000 sheets/year per practice 3D-printing technologies: −25% of supply chain waste

Abbreviations: percentage, “%”; kilograms carbon dioxide equivalents, “kgCO_2_e”; tonnes carbon dioxide equivalents, “TCO_2_e”; kilowatt-hour, “kWh”; three-dimensional, “3D”.

**Table 3:** Sustainable Dentistry: Strategies in Dental and Periodontal Practice.

Strategy Area	Key Actions in Dental Practice	Specific Actions in Periodontal Practice
Resource Efficiency (Energy and Water) [3,17,18,19,20,24,25,27]	- LED lighting, motion sensors, and energy-efficient HVAC - Solar panels and sustainability toolkits - Replace wet-ring vacuums, use recirculating autoclaves - Dental staff training on system-specific flushing protocols - PPE and clothing made from low-water-impact materials (e.g., bamboo, recycled plastic)	- Apply power-saving protocols during full-mouth disinfection and lengthy maintenance procedures - Renewable electricity for digital imaging and charting - Use low-flow devices during debridement and surgery - Perform flushing of suction lines, waterlines, handpieces, and turbines after each non-surgical or surgical periodontal procedure - Use anti-retraction valves
Air Pollution and Emissions Reduction [12,28,29,33]	- Amalgam separators - Low-toxicity disinfectants - Reduce emissions from anesthetic gases via gas scavenging/scrubbing systems - Optimize lab transport and appointment scheduling - Promote teledentistry to reduce travel emissions	- Minimize aerosol production via hand instrumentation during non-surgical periodontal therapy - Bundle short appointments (e.g., hygiene + check-up) - Adopt digital workflows and remote monitoring (e.g., intraoral cameras + teledentistry) - Restrict anesthetic gases to cases without alternatives
Recycling and Waste Management [33,34,39,40,41]	- Prefer sterilizable tools (steel tips, cloth gowns) - Multi-use gloves and recycling programs - Use color-coded bins - Staff training - Reduce incineration waste	- Use stainless steel periodontal instruments (e.g., stainless steel probes, ultrasonic tips) - Recycle implant packaging, barrier films - Reduce barrier films by reducing their sizes and choosing low-plastic surgical tools - Prefer cloth sterilization wrappings
Digitalization and Smart Technologies [3,17,38,46,47,49,50,53]	- Electronic health records, digital imaging - Cloud-based documentation and consent - Energy-efficient AI for diagnostics/planning - Apply explainable, energy-efficient AI - Prioritize energy-efficient AI models and hardware	- Digital periodontal charting and records - AI for disease staging, bone loss detection, and risk-based recall planning - Remote monitoring via apps and intraoral cameras to track clinical parameters - Create a large periodontal database for scientific research to minimize resource-intensive research
Education and Community Awareness [13,14,43,44,45,63]	- Integrate sustainability in curricula - Promote SDG-aligned training, community outreach, and patient education campaigns	- Integrate sustainable periodontics into training - Patient education on sustainable oral hygiene (e.g., avoiding microplastic-releasing mouthwashes/floss, recommending bamboo/replaceable interdental brushes)

Abbreviations: Light-Emitting Diode, “LED”; Heating Ventilation and Air Conditioning, “HVAC”; Personal Protective Equipment, “PPE”; Artificial Intelligence, “AI”; Sustainable Development Goals, “SDG”.
